# Características sociodemográficas asociadas a la prevalencia del consumo de tabaco en Costa Rica

**DOI:** 10.26633/RPSP.2020.17

**Published:** 2020-04-02

**Authors:** Azálea Espinoza Aguirre, Romain Fantin, Cristina Barboza Solis, Abraham Salinas Miranda

**Affiliations:** 1 Dirección de Vigilancia de la Salud Ministerio de Salud San José Costa Rica Dirección de Vigilancia de la Salud, Ministerio de Salud, San José, Costa Rica.; 2 Escuela de Salud Pública, Facultad de Medicina Universidad de Costa Rica Costa Rica Escuela de Salud Pública, Facultad de Medicina, Universidad de Costa Rica, Costa Rica.; 3 Facultad de Odontología Universidad de Costa Rica Costa Rica Facultad de Odontología, Universidad de Costa Rica, Costa Rica.; 4 Colegio de Salud Pública Universidad del Sur de la Florida Estados Unidos de América Colegio de Salud Pública, Universidad del Sur de la Florida, Estados Unidos de América.

**Keywords:** Uso de tabaco, adultos, determinantes epidemiológicos, análisis de regresión, Costa Rica, Tobacco use, epidemiologic factors, regression analysis, Costa Rica, Uso de tabaco, fatores epidemiológicos, análise de regressão, Costa Rica

## Abstract

**Objetivo.:**

Determinar las asociaciones existentes entre las características sociodemográficas y la prevalencia del consumo actual de tabaco en Costa Rica, según los resultados de la Encuesta Global de Tabaquismo en Adultos (GATS).

**Métodos.:**

Estudio epidemiológico, observacional de tipo transversal con representación nacional (n = 8607), que utilizó las variables sociodemográficas incluidas en la GATS realizada durante 2015. Se diseñó un modelo de regresión logística para predecir la influencia de esas variables en el consumo actual de tabaco. La variable dependiente es el consumo actual de tabaco considerando los determinantes sociales disponibles en la encuesta: género, nivel educativo, zona de residencia, edad y la composición del hogar.

**Resultados.:**

El modelo de regresión logística demuestra que ser mujer (OR = 0,29; *P* < 0,01), tener 65 años y más (OR = 0,61; *P* = 0,02), vivir en zona rural (OR = 0,63; *P* < 0,01) y vivir con otras personas (OR = 0,68; *P* < 0,01), en particular con niños de 15 años o menos (OR = 0,55; *P* < 0,01), son factores protectores del consumo de tabaco. El consumo de tabaco disminuye de forma significativa con el aumento de la riqueza medida por los artefactos en la casa en las mujeres, pero no en los hombres. Completar la educación secundaria es un factor protector en las personas de 15-34 años (OR = 0,47; *P* < 0,01), aunque no en las personas de 35 años y más.

**Conclusiones.:**

Existe una asociación entre las variables sociodemográficas disponibles en la encuesta GATS Costa Rica realizada en el 2015 y el consumo actual de tabaco. Intervenciones a nivel familiar y comunal podrían contribuir a que los consumidores abandonen el tabaquismo.

## Abstract (en)

**Objective.:**

To determine the associations between sociodemographic characteristics and the current prevalence of tobacco use in Costa Rica, based on the results of the Global Adult Tobacco Survey (GATS).

**Methods.:**

Cross-sectional observational epidemiological study, country-wide (n = 8607), that used the sociodemographic variables included in GATS 2015. A logistic regression model was designed to predict the impact of those variables on current tobacco use. The dependent variable is current tobacco use, considering the social determinants available in the survey: sex, educational level, area of residence, age, and household composition.

**Results.:**

The logistic regression model shows that being female (OR = 0.29; *P* < 0.01), being 65 years old and over (OR = 0.61; *P* = 0.02), living in a rural area (OR = 0.63; *P* < 0.01), and living with other people (OR = 0.68; *P* < 0.01), in particular with children 15 years old or under (OR = 0.55; *P* < 0.01), are protective factors against tobacco use. Tobacco use declines significantly with increased wealth, as measured by household items, in women but not in men. Completing secondary education is a protective factor in people 15-34 years old (OR = 0.47; *P* < 0.01) but not in people 35 and over .

**Conclusions.:**

There is an association between the sociodemographic variables found in the GATS Costa Rica survey carried out in 2015 and current tobacco use. Interventions at the family and community levels could help consumers give up smoking.

## RESUMO

**Objetivo.:**

Determinar as associações existentes entre as características sociodemográficas e a prevalência do consumo presente de tabaco na Costa Rica, segundo os resultados da Pesquisa Global sobre Tabagismo em Adultos (*Global Adult Tobacco Survey* - GATS).

**Métodos.:**

Trata-se de um estudo epidemiológico observacional transversal com representatividade nacional (n = 8.607) com o uso das variáveis sociodemográficas estudadas na GATS realizada em 2015. Usou-se um modelo de regressão logística para predizer a influência das variáveis estudadas no consumo presente de tabaco. A variável dependente foi o consumo presente de tabaco levando em consideração os determinantes sociais disponíveis na pesquisa: gênero, nível de escolaridade, área de residência, idade e composição do domicílio.

**Resultados.:**

Observou-se, no modelo de regressão logística, que ser do sexo feminino (OR 0,29; *P* < 0,01), ter 65 anos ou mais (OR 0,61; *P* = 0,02), residir na zona rural (OR 0,63; *P* < 0,01) e viver em um domicílio com outras pessoas (OR 0,68; *P* < 0,01), sobretudo com crianças menores de 15 anos (OR 0,55; *P* < 0,01), são fatores de proteção contra o consumo de tabaco. O consumo de tabaco diminui de forma significativa com o aumento da renda (medida de acordo com o número de serviços e utilidades domésticas) apenas entre as mulheres. Ter o ensino médio completo é um fator de proteção na faixa etária entre 15 e 34 anos (OR 0,47; *P* < 0,01), mas não entre as pessoas acima de 35 anos.

**Conclusões.:**

Existe uma associação entre as variáveis sociodemográficas estudadas na GATS de 2015 e o consumo presente de tabaco na Costa Rica. Intervenções realizadas ao nível da família e da comunidade poderiam contribuir para a cessação do tabagismo.

El consumo de tabaco es todavía un reto en términos de salud pública, especialmente para los países de renta baja y media (1, 2). Estimaciones realizadas por el estudio de la carga global de enfermedades mencionan que estos países presentan un aumento general en la prevalencia del consumo de tabaco (2), con implicaciones sociales y económicas importantes (1). La Organización Mundial de la Salud (OMS) considera el consumo del tabaco como prioridad de salud pública, pues existen inequidades sociales tanto entre los países como en su interior (3, 4). Estas inequidades sociales no solo afectan a una buena parte de la población, sobre todo en países de ingresos medios y altos, sino que además están en aumento (5).

Se estima que aproximadamente 80% de los fumadores viven en países de ingresos bajos o medios, donde la carga de morbilidad y mortalidad asociada al tabaco es mayor. Por otro lado, los consumidores de tabaco que mueren en forma prematura privan a sus familias de ingresos, aumentan el costo de la atención sanitaria y dificultan el desarrollo económico (6, 7).

El tabaquismo es una adicción compleja que posee varios determinantes, entre los cuales, los principales identificados son físicos, sociales y psicosociales. Estos dos últimos representan un eje de análisis de las inequidades sociales en el consumo de tabaco. Como determinantes sociales identificados, se mencionan en particular aquellos asociados a la posición socioeconómica (nivel de escolaridad, ocupación, empleo e ingresos) (8). La evidencia sugiere la existencia de un gradiente social continuo entre todos los estratos sociales, en el que las personas provenientes de las poblaciones socioeconómicas más bajas son aquellas que poseen mayores posibilidades de consumir tabaco cuando se las compara con el siguiente grupo social más aventajado, y así a lo largo de toda la jerarquía social (5, 8, 9). Estudios internacionales mencionan que las tasas de tabaquismo son particularmente altas entre las personas que llevan mucho tiempo desempleadas, las personas sin hogar, las que tienen enfermedades mentales, los privados de libertad, los padres solteros y algunos grupos de nuevos inmigrantes y minorías étnicas (10).

Con respecto a los determinantes psicosociales, un autovalimiento reducido (11) y autoestima (12), así como el aislamiento social, son factores asociados al inicio del consumo del tabaco e influyen en la cesación del consumo de tabaco (13).

Durante el 2015, el Ministerio de Salud de Costa Rica (MS) realizó la Encuesta de Consumo Global de Tabaquismo en Adultos (GATS por sus siglas en inglés: *Global Adults Tobacco Survey*), que es el estándar mundial para el monitoreo sistemático del consumo de tabaco en personas adultas (fumadoras y no fumadoras) de 15 y más años. Los temas tratados en la encuesta fueron: la prevalencia del consumo de tabaco (fumado y sin humo), la exposición al humo de tabaco ajeno y políticas relacionadas, el abandono del tabaquismo, los conocimientos, las actitudes y percepciones, y la exposición a los medios y economía. Con los resultados se elaboró y publicó una hoja resumen y un resumen ejecutivo (14). Los resultados de la primera GATS demostraron que Costa Rica ha puesto en marcha políticas de control del tabaco y que estas han sido exitosas (15).

Se sabe que el consumo de tabaco es una práctica muy condicionada por el género (16), por lo que se hace necesaria la integración del enfoque de género en la investigación del consumo y la cesación del tabaquismo en Costa Rica. Los roles de género son construcciones sociales que que conforman los comportamientos, las actividades, las expectativas y las oportunidades que se consideran apropiados en un determinado contexto sociocultural para todas las personas. Además, la perspectiva de género hace referencia a las relaciones desiguales entre las personas con diferente género y a la distribución del poder en esas relaciones (17).

Los factores socioeconómicos y demográficos como la edad, la educación y el empleo son considerados determinantes sociales de la salud de tipo estructural (12). Son llamados estructurales o de posición social porque estos reflejan las diferencias de riqueza, prestigio y poder en las sociedades. Estos influyen en las circunstancias directas en que las personas nacen, crecen, viven, trabajan y envejecen, incluido el sistema de salud (18).

El objetivo de esta investigación es determinar las asociaciones existentes entre las características sociodemográficas y la prevalencia del consumo actual de tabaco en Costa Rica, según los resultados de la encuesta GATS.

## MÉTODOS

Es un estudio epidemiológico, observacional de tipo transversal con representación nacional. GATS Costa Rica 2015 se realizó mediante el diseño un muestreo probabilístico en tres etapas: selección proporcional al tamaño de las unidades primarias de muestreo (UPM), selección sistemática de viviendas y la selección aleatoria de los adultos. La representación a nivel nacional se obtuvo con dos tipos de conglomerados: por sexo y por zona de residencia (urbana y rural) (14). El tamaño de la muestra se calculó tomando en cuenta la prevalencia del consumo de tabaco (14,4% en el año 2010 ) (19) y se fijaron un error en las estimaciones de 3,0%, un nivel de confianza de 95% y un efecto de diseño de 2,0. GATS fue diseñada para dos grupos de análisis: por residencia y género (20). La muestra final ajustada con la no respuesta fue de 9 600 individuos. Se visitaron 4 850 viviendas en el área urbana y 4 830 en la rural (14). Para este estudio, se utilizó la base de datos de GATS adaptada a Costa Rica y las variables del cuestionario también adaptado a Costa Rica (14). En el cuadro 1 se resumen las principales características de la muestra.

La variable dependiente fue el consumo del tabaco y se utilizó la pregunta: “¿Fuma usted actualmente tabaco de forma diaria, menos frecuente que a diario o del todo, no fuma actualmente?”. Esta variable se recodificó como dicotómica o binaria, para lo que se consideraron como fumadores las personas que respondieron “Diariamente” o “Menos que diariamente”; y no fumadores las personas que respondieron “Del todo, no fuma actualmente”.

Se analizaron las variables socioeconómicas y demográficas de GATS para identificar grupos de población que se diferencien en cuanto al consumo de tabaco. Las variables independientes fueron la edad (en años categorizada), el sexo (masculino/femenino), composición del hogar (persona sola y varias personas adultas/presencia de menores de 15 años), y zona de residencia (urbana/rural). Además, se incluyeron dos variables para medir la posición socioeconómica (21): el nivel de educación (completó la educación secundaria/no) y artefactos en la casa (favorecido, intermedio y desfavorecido). Los artefactos corresponden a una serie de 11 preguntas sobre los servicios de la casa: electricidad, teléfono y carro, entre otros. Se unieron las respuestas de carro y motocicleta. Los hogares que tenían nueve artefactos o más se clasifican como “Favorecido”, los que tenían 7 o 8 artefactos como “Intermedio” y 6 artefactos o menos como “Desfavorecido”.

La relación entre las variables independientes y el consumo de tabaco se analizó con las ponderaciones calculadas por la GATS, con el propósito de obtener una muestra representativa de la población de Costa Rica. En el cuadro 1 se presenta la relación bivariada ponderada entre cada variable y el consumo de tabaco. Se utilizó la función *svy* del programa STATA^®^ para calcular la estadística F (*design-based F*) y la significancia estadística (*P* < 0,05). En el cuadro 2 se 2 presentan los resultados del modelo de regresión logística ajustados por todas las covariables. Como medida de asociación, se realizó la exponenciación del coeficiente beta que equivale a la razón de momios o conocida también como *odds-ratio* (OR).

Se implementaron un análisis estratificado por sexo y un análisis por edad para confirmar la robustez de los resultados y evidenciar efectos de moderación de dichas variables en la variable dependiente. El análisis por edad se hizo dividiendo la muestra ponderada en dos grupos iguales, mediante uso de la edad quinquenal. Se dividió la muestra entre la población de 34 años y menos (45,3% de la muestra) y la población de 35 años y más (54,7%).

**CUADRO 1.** Descripción de las características de la muestra: frecuencias absolutas (N), relativas (%), relativas ponderadas (%P) y consumo de tabaco

Características	N	%	%*P*	Consumo de tabaco (%)
Total	8607			8,9
Sexo				*P* < 0,01
Masculino	3544	41,2	50,3	13,4
Femenino	5063	58,8	49,7	4,4
Edad (años)				*P* < 0,01
15-24	1377	16,0	23,3	7,2
25-44	3049	35,4	40,9	9,8
45-64	2662	30,9	26,5	10,4
≥ 65	1519	17,7	9,3	5,3
Zona				*P* < 0,01
Urbana	4257	49,5	74,0	9,6
Rural	4350	50,5	26,0	7,1
Educación				*P* < 0,05
Completó nivel secundario	2306	72,8	65,3	7,5
No completó nivel secundario	6264	26,8	34,1	9,6
NR	37	0,4	0,7	NC
Artefactos en la casa (riqueza)				*P* > 0,05
Favorecido	3623	42,1	52,9	8,6
Intermedio	3883	45,1	39,1	9,2
Desfavorecido	1101	12,8	8,0	9,8
Composición del hogar				
Persona sola	1330	15,5	6,0	12,7
Varias personas adultas	3056	35,5	43,6	9,6
Presencia de menores de 15 años	4221	49,0	50,5	8,0

NR: no responde.

***Fuente:*** elaboración propia a partir de datos de la investigación.

Los modelos se estratificaron por sexo y grupos de edad para evidenciar el peso de cada determinante, y se requiere que las mismas variables estén incluidas en los diferentes modelos.

El estudio de GATS fue considerado categoría exenta de revisión por el comité institucional de ética del Ministerio de Salud de Costa Rica, por lo que para este análisis no se requirió elaborar un consentimiento informado y por ser un análisis secundario de bases de datos públicas.

## RESULTADOS

GATS Costa Rica es una encuesta de representación nacional, por lo que los resultados de este estudio pueden generalizarse a toda la población. En el cuadro 1 se presentan los resultados del análisis bivariado entre el consumo de tabaco y las características socioeconómicas y demográficas. Los hombres fuman más que las mujeres (13,4% frente a 4,4% respectivamente, *P* < 0,01). De igual manera, existe una relación significativa entre la edad y el consumo de tabaco, donde los que más fuman son los que tienen 45-64 años. Las personas que viven en la zona rural fuman menos que las personas que viven en zona urbana (7,1% frente a 9,6%, *P* < 0,01). Los que completaron la educación secundaria fuman menos que las personas que no la completaron (7,5% frente 9,6%). No existe una relación significativa entre el número de artefactos en la casa y el consumo de tabaco. Por último, la composición del hogar guarda relación con el consumo de tabaco.

**CUADRO 2.** Análisis mutivariado de la relación entre el consumo de tabaco y las características sociodemográficas (modelo de regresión logística, N = 8607)

Características sociodemográficas	OR	IC95%	*P*^a^
Sexo			
Hombres	1		
Mujeres	0,29	(0,23-0,36)	< 0,01
Edad (años)			
15-24	1		
25-44	1,46	(1,07-1,99)	0,02
45-64	1,43	(1,04-1,96)	0,03
≥ 65	0,61	(0,40-0,93)	0,02
Zona			
Urbana	1		
Rural	0,63	(0,51-0,77)	< 0,01
Educación			
Completó nivel secundario	1		
No completó nivel secundario	1,39	(1,06-1,82)	0,02
No resoponde			
Artefactos en la casa (riqueza)			
Favorecido	1		
Intermedio	1,17	(0,92-1,50)	0,21
Desfavorecido	1,3	(0,91-1,86)	0,15
Composición del hogar			
Persona sola	1,83	(1,40-2,40)	< 0,01
Varias personas adultas	1,25	(0,99-1,59)	0,07
Presencia de menores de 15 años	1		

OR, *odds ratio*; IC95%, intervalo de confianza de 95%.

a Valor de *P*: prueba z de regresión logística.

***Fuente:*** elaboración propia a partir de datos de la investigación.

Los resultados del análisis multivariado entre el consumo de tabaco y las características sociodemográficas se presentan en el cuadro 2. El modelo de regresión logística confirma los resultados del análisis bivariado. Ser mujer, tener menos de 25 años o 65 años y más, vivir en zona rural, haber completado la educación secundaria, y vivir con otras personas y en particular con niños de 15 años o menos son factores protectores del consumo de tabaco. No existe una relación significativa entre la riqueza medida por los artefactos en la casa y el consumo de tabaco.

El análisis por sexo (cuadro 3) confirmó los resultados presentados antes con respecto a la relación entre el consumo de tabaco y la edad y la zona de residencia. El análisis en los hombres también confirmó la relación entre el consumo de tabaco y el nivel de educación o composición del hogar, y la ausencia de relación entre el consumo de tabaco y la riqueza. En las mujeres, no existía relación entre el consumo de tabaco y la composición del hogar o el nivel de educación (OR = 1,03; no significativo [NS]). Sin embargo, el consumo de tabaco de las mujeres aumentaba en los hogares intermedios (OR = 1,56; *P* = 0,04) y desfavorecidos (OR = 2,09; *P* = 0,03) en comparación con los hogares favorecidos.

El análisis por edad (cuadro 4) confirmó los resultados presentados antes con respecto a la relación entre el consumo de tabaco y el sexo, la zona de residencia y la composición del hogar. En los dos análisis, no se encontró una relación entre la riqueza y el consumo de tabaco. El análisis en las personas de 34 años y menos evidenció una relación fuerte entre el nivel de educación y el consumo de tabaco (OR = 2,04; *P* < 0,01). Por el contrario, no se evidenció una relación significativa entre el nivel de educación y el consumo de tabaco en los adultos de 35 años y más (OR = 0,93; NS).

**CUADRO 3.** Análisis mutivariado de la relación entre el consumo de tabaco y las características sociodemográficas por sexo (modelo de regresión logística, N = 8607)

Características sociodemográficas	Hombres			Mujeres		
	OR	IC95%	*P*^a^	OR	IC95%	*P*^a^
Edad (años)						
15-24	1			1		
25-44	1,37	(0,94-1,98)	0,10	1,78		0,04
45-64	1,35	(0,93-1,98)	0,12	1,75		0,05
≥ 65	0,56	(0,34-0,91)	0,02	0,81		0,62
Zona						
Urbana	1			1		
Rural	0,71	(0,56-0,90)	< 0,01	0,42		< 0,01
Educación						
Completó nivel secundario	1			1		
No completó nivel secundario	1,54	(1,11-2,15)	0,01	1,03		0,88
Artefactos en la casa (riqueza)						
Favorecido	1			1		
Intermedio	1,06	(0,78-1,43)	0,71	1,56		0,04
Desfavorecido	1,11	(0,73-1,67)	0,63	2,09		0,03
Composición del hogar						
Persona sola	2,03	(1,46-2,83)	< 0,01	1,40		0,16
Varias personas adultas	1,31	(0,98-1,76)	0,07	1,08		0,69
Presencia de menores de 15 años	1			1		

OR, *odds ratio*; IC95%, intervalo de confianza de 95%.

a Valor de *P*: prueba z de regresión logística.

***Fuente:*** elaboración propia a partir de datos de la investigación.

**CUADRO 4.** Análisis mutivariado de la relación entre el consumo de tabaco y las características sociodemográficas por edad (modelo de regresión logística, N = 8607)

Características sociodemográficas	15-34 años			35 años y más		
	OR	IC95%	*P*^a^	OR	IC95%	*P*^a^
Sexo						
Hombres	1			1		
Mujeres	0,27	(0,19-0,38)	< 0,01	0,31	(0,24-0,41)	< 0,01
Zona						
Urbana	1			1		
Rural	0,69	(0,50-0,97)	0,03	0,61	(0,47-0,78)	< 0,01
Educación						
Completó nivel secundario	1			1		
No completó nivel secundario	2,04	(1,33-3,14)	< 0,01	0,93	(0,66-1,30)	0,66
Artefactos en la casa (riqueza)						
Favorecido	1			1		
Intermedio	1,19	(0,80-1,78)	0,40	1,16	(0,85-1,58)	0,36
Desfavorecido	1,68	(0,95-2,99)	0,08	1	(0,64-1,55)	1,00
Composición del hogar						
Persona sola	2,44	(1,47-4,07)	< 0,01	1,45	(1,07-1,97)	0,02
Varias personas adultas	1,40	(0,96-2,04)	0,08	1,07	(0,80-1,43)	0,66
Presencia de menores de 15 años	1			1		

OR, *odds ratio*; IC95%, intervalo de confianza de 95%.

a Valor de *P*: prueba z de regresión logística.

***Fuente:*** elaboración propia a partir de datos de la investigación.

## DISCUSIÓN

Los principales resultados de este estudio muestran la asociación entre las variables socioeconómicas y demográficas disponibles en la encuesta GATS Costa Rica y el consumo de tabaco. Ser mujer, tener 65 años y más, habitar en zona rural, y vivir con otras personas en el hogar son factores protectores del consumo de tabaco. En términos socioeconómicos, el consumo de tabaco disminuye de manera significativa con el aumento de la riqueza (medida con la disponibilidad de artefactos en el hogar) solo en el caso de las mujeres. Completar la educación secundaria es un factor protector para las personas entre 15 y 34 años y más, pero no para las personas mayores de 35 años.

Estos resultados son similares a los descritos en la literatura nacional (15), internacional y, en particular, con los reportados por las encuestas GATS realizadas en otros países. Con respecto a las diferencias de sexo, estas también han sido descritas en Costa Rica desde 1990 (22). Reportes previos de todo el mundo indican que la prevalencia de tabaquismo es mayor en hombres en todos los países estudiados (23). Sin embargo, las diferencias de prevalencia por sexo varían según los países; las encuestas GATS muestran diferencias de prevalencia muy importantes de consumo por sexo en China, India, China, Egipto o Vietnam, mientras que en el Reino Unido o en los Estados Unidos son menos marcadas (23). En este sentido, los resultados en Costa Rica sitúan al país entre estas dos tendencias. Estas diferencias por sexo poseen explicaciones diversas y complejas, en general relacionadas con el estatus y el significado social del consumo de tabaco. Según la OMS, el sexo aún es en una gran cantidad de países, el principal predictor del uso de tabaco, y se estima que 80% de las muertes asociadas a los hombres en el mundo suceden en países de renta baja y media (24). La adopción de comportamientos de riesgo, como el consumo de tabaco y de alcohol por parte de los hombres, responde a la construcción de la masculinidad y lo que significa “ser hombre” en cada cultura, así como a las conductas que se les atribuyen (25). Esta construcción de la masculinidad exalta el consumo de tabaco (sobre todo fumado) en los hombres y lo denigra en las mujeres (25), situación que se reprodujo durante años en la publicidad del tabaco (26). Se sugieren entonces explicaciones relacionadas con la equidad de género en los países , el estatus socioeconómico y los procesos de difusión y normalización del tabaco en la sociedad (27).

La edad es un determinante común del consumo de tabaco en la mayoría de los países, donde los adultos en edad mediana fuman más que los adultos jóvenes y los adultos mayores (23). Esto podría explicarse, de manera parcial, como un efecto de las políticas y medidas tomadas en el país, en la aplicación de la Ley 9028: Ley General de Control de Tabaco y sus Efectos Nocivos en la Salud y que pudieron ser más efectivas en la población joven que en los adultos fumadores.

Este estudio evidenció una relación compleja entre posición socioeconómica y consumo de tabaco. Al igual que otras investigaciones en América Latina (28), se demostró que hay mayor prevalencia de tabaquismo en las zonas urbanas en comparación con las zonas rurales.

Las poblaciones de la zona rural tienen ingresos e indicadores socioeconómicos mucho más bajos que las de la zona urbana (21). Este resultado es también conforme a otros estudios en Costa Rica, que evidenciaron que la prevalencia más alta se dio en la región central, la zona más urbanizada del país (22). Sin embargo, este hallazgo no se confirma en otros países occidentales. En Estados Unidos se han observado diferencias entre las zonas urbanas y rurales, con las poblaciones rurales más propensas al consumo de tabaco (29). Otros estudios en países de renta baja, como Bangladesh y Nigeria también muestran que las zonas rurales son aquellas que presentan prevalencias más altas (30). Esto puede explicarse por las disparidades de nivel de educación y socioeconómico encontradas en esos países. En el caso de Costa Rica, se podría especular que la diferencia del nivel del desarrollo y el retardo en la adopción de los estilos de vida occidentales podrían explicar por qué las poblaciones rurales presentan una menor prevalencia de consumo de tabaco; esto hace que su transición epidemiológica haya sido más lenta comparada a las zonas urbanas (31). Otros estudios son necesarios para entender el importante rol protector de la zona rural.

Este estudio también mostró que el consumo de tabaco está asociado con el nivel de educación en los 15-34 años, así como con el nivel de riqueza en las mujeres, como ocurre en otros países (32); en ambos casos, las personas más desfavorecidas tienen una prevalencia superior en comparación con a las personas más favorecidas. Este resultado está en contradicción con estudios previos en Costa Rica, en los cuales el consumo de tabaco se asociaba con ingresos mayores (22). La diferencia de consumo según el nivel de educación es particularmente importante en los jóvenes, lo que plantea inquietudes sobre el aumento de las inequidades sociales de salud en el país.

La literatura internacional refiere que el nivel de educación es un reflejo de los recursos materiales, sociales, culturales e intelectuales (33). Esto permite a las personas con un buen capital educativo ser más receptivos a los mensajes de prevención de las enfermedades, les permite comunicarse mejor con el personal de salud y tener una mejor relación con su cuerpo y las representaciones propias de la salud, así como entender de mejor manera la forma de acceder a los servicios de salud (33). Con relación a esto, solo se pusieron en evidencia diferencias sociales por nivel de educación en un grupo de edad (los menores de 35 años) y por nivel de riqueza, solo en mujeres. La riqueza en sí misma no evita consumir tabaco, sino su conversión en recursos que potencialmente mejoran la salud, como el acceso a servicios de salud, actividades de ocio y el deporte (33). Estos, a su vez, son determinantes tanto del consumo como de la cesación del tabaco (34). Encontrar un efecto del nivel de educación en las personas más jóvenes es preocupante, pues podría revelar un inicio de brecha social, lo que indica que las inequidades sociales en consumo de tabaco podrían aumentar en los próximos años.

En los resultados de esta investigación, se identificó que el hecho de vivir con menores de 15 años es un factor protector, lo que puede poseer explicaciones tanto sociales (prevenir el fumado pasivo en menores) como psicosociales (mayor apoyo social) (13).

Este estudio presenta varias limitaciones, ya que no se incorporaron en el análisis todos los determinantes sociales descritos en la literatura, en particular los factores psicosociales, pues no se encontraban disponibles en la encuesta GATS. Entre los determinantes psicosociales reportados se incluyen las estrategias de afrontamiento psicosociales ante situaciones adversas y estresantes (*coping*) durante la vida (35), así como la autoeficacia, autoestima, el apoyo y el aislamiento social (personas en situación de calle, indigencia, o abandono) (8). Este estudio se enfocó en los determinantes sociales del consumo actual de tabaco en la población de 15 y más anos, lo cual impedía incluir las variables que describen los patrones de consumo (la edad de inicio y la adicción al tabaco) debido a que no son determinantes sociales, sino mecanismos que ligan estos con el consumo de tabaco. El poder estadístico del análisis no permitió utilizar un modelo con interacciones que hubiera permitido demostrar con más fuerza las diferencias por sexo y edad. Por último, el país no cuenta con datos locales que describan el cumplimiento de las diferentes políticas de control de tabaco, lo que impidió ver su efecto sobre el consumo.

A pesar de estas limitaciones, este estudio presenta fuerzas. Se trata de una encuesta nacional, representativa de la población, cuya muestra sobrepasa las 8 000 observaciones. Esto permitió realizar análisis robustos, estratificados por sexo y por edad. La metodología utilizada basada en GATS ha sido estandarizada a nivel internacional, lo que permite realizar comparaciones con los otros países que la utilizaron.

Se analizaron factores socioeconómicos que no habían sido estudiados antes, como la riqueza y la composición del hogar. Se utilizó un modelo de regresión logística, lo que permite desagregar el efecto de cada variable independiente. En este modelo, al contrario de los análisis descriptivos antes publicados (15), se tienen en cuenta las correlaciones existentes entre cada determinante (área, nivel de educación, riqueza, entre otros). En este sentido, aportó informaciones novedosas sobre el rol exacto de cada determinante sobre el consumo de tabaco en Costa Rica.

El país ha realizado esfuerzos exitosos para reducir consumo el tabaco: la prevalencia disminuyó de 14,3% (19) a 8,9% (14) en un período de 25 años. Sin embargo, existen retos vigentes con respecto a las poblaciones con mayores riesgos. Este estudio permite identificar algunos determinantes socioeconómicos y demográficos del consumo de tabaco, que puede representar un primer paso para continuar los estudios por grupos de población, desde un enfoque de equidad en salud.

A nivel individual, desde la perspectiva de la prevención de las enfermedades, se debe sensibilizar al personal de clínicas de cesación de fumado y exhortarlos a tomar en cuenta el contexto social de las personas para aumentar las probabilidades de éxito.

Como conclusiones deben destacarse las siguientes: primera, el estudio logró determinar que existe asociación entre las variables sociodemográficas incluidas en la encuesta GATS Costa Rica realizada en 2015 y la prevalencia de consumo actual de tabaco. Segunda, el apoyo familiar fue un factor protector; este aspecto debe ser tenido en cuenta para implementar programas de intervención a nivel familiar y comunal, y lograr así que más consumidores abandonen el hábito de fumar. Tercero, existen inequidades sociales de salud en el consumo de tabaco donde los jóvenes con alto nivel educativo son los favorecidos.

### Contribución de los autores.

Todos los autores han participado en el diseño del estudio original, en la planificación, en el análisis de los datos, en la interpretación de los resultados y en la redaccióndel manuscrito. Todos los autores revisaron y aprobaron la versión final.

### Declaración.

Las opiniones expresadas en este manuscrito son responsabilidad del autor y no reflejan necesariamente los criterios ni la política de la *RPSP/PAJPH* y/o de la OPS.
